# Lifespan extension conferred by mitogen-activated protein kinase kinase kinase 5 (*MAP3K5*) longevity-associated gene variation is confined to at-risk men with a cardiometabolic disease

**DOI:** 10.18632/aging.202844

**Published:** 2021-03-19

**Authors:** Brian J. Morris, Randi Chen, Timothy A. Donlon, Kamal H. Masaki, D. Craig Willcox, Richard C. Allsopp, Bradley J. Willcox

**Affiliations:** 1 Department of Research, Kuakini Medical Center, Honolulu, HI 96817, USA; 2 Department of Geriatric Medicine, John A. Burns School of Medicine, University of Hawaii, Honolulu, HI 96813, USA; 3 School of Medical Sciences, University of Sydney, New South Wales, Australia; 4 Department of Cell and Molecular Biology and Department of Pathology, John A. Burns School of Medicine, University of Hawaii, Honolulu, HI 96813, USA; 5 Department of Human Welfare, Okinawa International University, Okinawa, Japan; 6 Institute for Biogenesis Research, University of Hawaii, Honolulu, HI 96822, USA

**Keywords:** lifespan, genetics, diabetes, hypertension, coronary heart disease

## Abstract

Genetic variants of the kinase signaling gene *MAP3K5* are associated with longevity. Here we explore whether the longevity-association involves protection against mortality in all individuals, or only in individuals with aging-related diseases. We tested the strongest longevity associated single nucleotide polymorphism (SNP), *rs2076260,* for association with mortality in 3,516 elderly American men of Japanese ancestry. At baseline (1991–1993), 2,461 had either diabetes (n=990), coronary heart disease (CHD; n=724), or hypertension (n=1,877), and 1,055 lacked any of these cardiometabolic diseases (CMDs). The men were followed from baseline until Dec 31, 2019. Longevity-associated genotype *CC* in a major allele homozygote model, and *CC*+*TT* in a heterozygote disadvantage model were associated with longer lifespan in individuals having a CMD (covariate-adjusted hazard ratio [HR] 1.23 [95% CI: 1.12–1.35, *p=*2.5x10^–5^] in major allele homozygote model, and 1.22 [95% CI: 1.11–1.33, *p=*1.10x10^–5^] in heterozygote disadvantage model). For diabetes, hypertension and CHD, HR *p*-values were 0.019, 0.00048, 0.093, and 0.0024, 0.00040, 0.0014, in each respective genetic model. As expected, men without a CMD outlived men with a CMD (*p*=1.9x10^–6^). There was, however, no difference in lifespan by genotype in men without a CMD (*p*=0.21 and 0.86, respectively, in each genetic model). In conclusion, we propose that in individuals with a cardiometabolic disease, longevity-associated genetic variation in *MAP3K5* enhances resilience mechanisms in cells and tissues to help protect against cardiometabolic stress caused by CMDs. As a result, men with CMD having longevity genotype live as long as all men without a CMD.

## INTRODUCTION

Mitogen-activated protein kinase kinase kinase 5 (MAP3K5, also termed ASK1 [apoptosis signal-regulating kinase 1]) is a member of a family of enzymes involved in kinase signaling cascades in the cell. MAP3K5 plays an important role in cellular responses evoked by changes in environment. These include cell differentiation and survival, apoptosis, innate immune response, and oxidative stress response [1]. It mediates signal transduction of oxidative stress and receptor-mediated inflammatory signals, such as ones involving tumor necrosis factor and lipopolysaccharide. Its crucial role in the apoptosis signal transduction pathway is mediated by mitochondria-dependent caspase activation. MAP3K5 plays a role in the pathology of a wide range of diseases in which reactive oxygen species (ROS) and/or endoplasmic reticulum stress are causative factors. MAP3K5/ASK1 may influence *in vivo* insulin action and obesity, and MAP3K5 variants are associated with type 2 diabetes [2]. MAP3K5 may prevent stress-induced disorders and protect from bacterial and viral infection under physiological circumstances. On the contrary, it may exert adverse effects through excessive cellular apoptosis and increased inflammation under some pathological conditions, such as present in neurodegenerative disorders, cardiovascular diseases, inflammatory diseases, and chronic inflammation-induced carcinogenesis [3]. Once activated, MAP3K5/ASK1 acts as an upstream activator of the MKK/JNK and p38 MAPK signal transduction cascades through phosphorylation and activation of several MAP kinase kinases such as MAP2K4/SEK1, MAP2K3/MKK3, MAP2K6/MKK6 and MAP2K7/MKK7. These in turn activate p38 MAPKs and c-jun N-terminal kinases (JNKs). Both p38 MAPK and JNKs control the transcription factor activator protein-1 (AP-1) [4]. While the above pathways are critical for senescence, aging, and age-associated cardiovascular diseases MAP3K5 might not be the cause, but rather a response [5, 6].

In a study of 33 single nucleotide polymorphisms (SNPs) of *MAP3K5* in our cohort of American men of Japanese ancestry (Supplementary Methods, Supplementary Tables 1, 2), alleles of two adjacent SNPs, and haplotypes of these alleles, were associated with longevity in men aged ≥ 95 years [7]. Bonferroni corrected *p*-values were 0.0043 for the SNP *rs2076260* and 0.032 for the SNP *rs6904753* in a heterozygous disadvantage model (Supplementary Table 3). Haplotype analysis of these yielded a *p*-value of 0.00004. Our rationale for studying *MAP3K5* was because in mouse liver *Map3k5* was differentially expressed in response to caloric restriction [8], a well-known longevity enhancer.

In the present study we tested the hypothesis that the longevity-associated alleles of *MAP3K5* SNP *rs2076260* mediate their effect on lifespan at least in part by protection against the detrimental effects of aging-related cardiometabolic diseases (CMD), namely diabetes and/or hypertension and/or coronary heart disease (CHD).

## RESULTS

### Characteristics of subjects

Baseline (1991–1993) characteristics of men in the study for each genotype of *rs2076260*, adjusting, for age, genotype, and prevalence of medical conditions are shown in Supplementary Table 4. By December 31, 2019, 3,480 out of 3,516 subjects had died during the 29 years of follow-up. At baseline, among the 3,516 participants, 29% had been diagnosed with diabetes alone, 53% with hypertension alone, 21% with CHD alone, and 14% with cancer alone. Seventy percent had at least one CMD, and 5.2% had all three CMDs. Mean age at death was 88.6 ± 6.0 years for men with at least one CMD, and 89.4 ± 6.0 years for men who did not have any of the CMDs (*p* < 0.0001).

### *MAP3K5* genotype and survival in men with a CMD and men without a CMD

**Figure 1:**
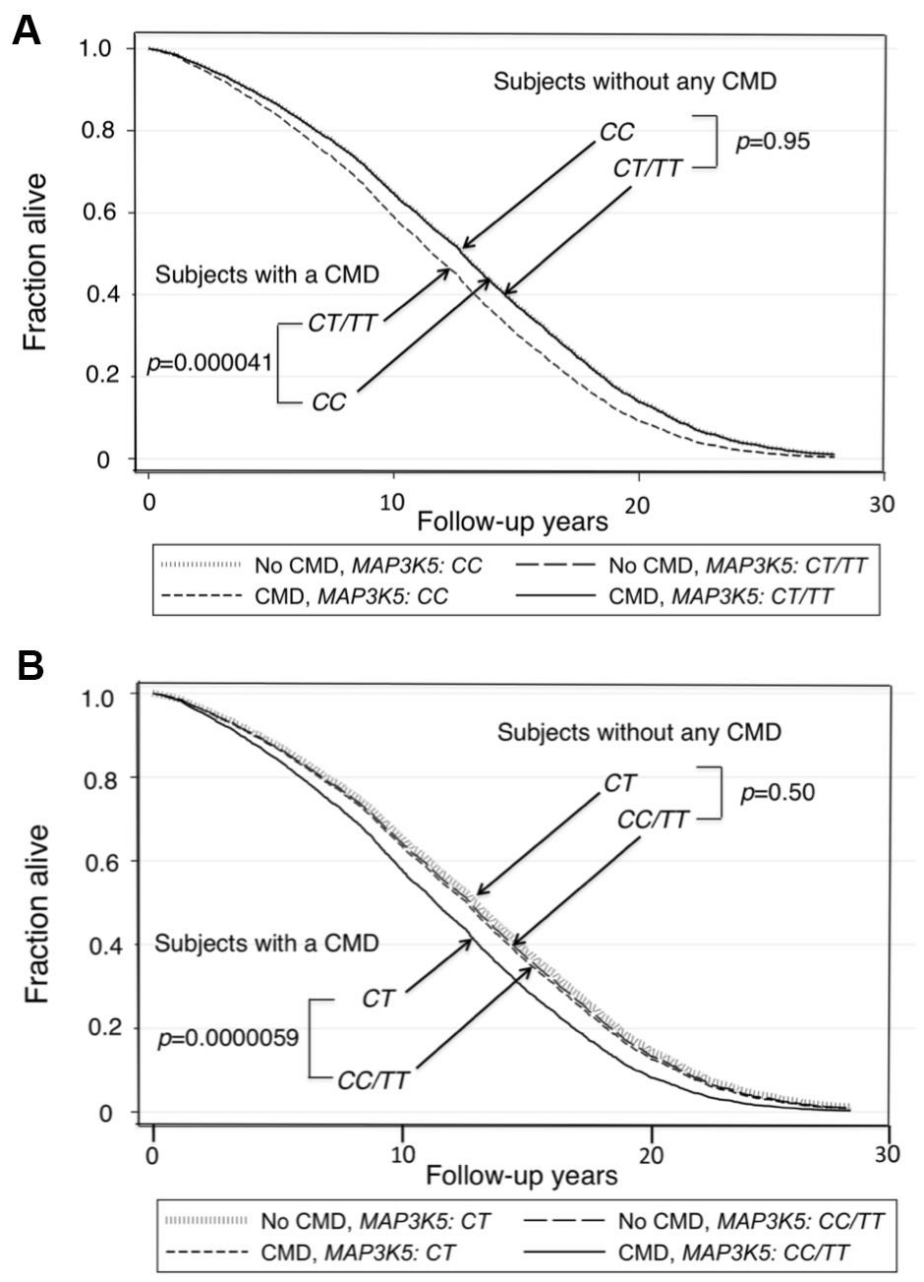
**Survival curves spanning the period from baseline (1991–1993) to Dec 31, 2019 for men with and men without a CMD according to genotypes of *MAP3K5* SNP *rs2076260*.** The survival probabilities were estimated from the Cox proportional hazard model: h(t) = h(t0) * exp(β1*Age + β2*BMI + β3*Glucose + β4*CMD + β5*MAP3K5_xx + β6* (CMD*MAP3K5_xx)), where “xx” is genotype, by fixing age at 75 years, BMI at the mean, 23.5 kg/m^2^, and glucose at the mean, 113 mg/dL (where β6 is the effect of the interaction of CMD with *MAP3K5* genotype on mortality, for *CC* vs *CT*/*TT*, i.e., a recessive model, giving *p*(β6) = 0.023). (**A**) Survival curves for men with a CMD vs. men without a CMD for major allele homozygote (*CC*) vs. minor allele carriers (*CT*+*TT*) (*p*=0.000041 and *p*=0.95, respectively). (**B**) Survival curves for men with a CMD vs. men without a CMD for heterozygote disadvantage model, *CT* vs. *CC/TT* (*p*=0.0000059 and *p*=0.50, respectively, giving *p*(β6) = 0.057).

**Figure 2:**
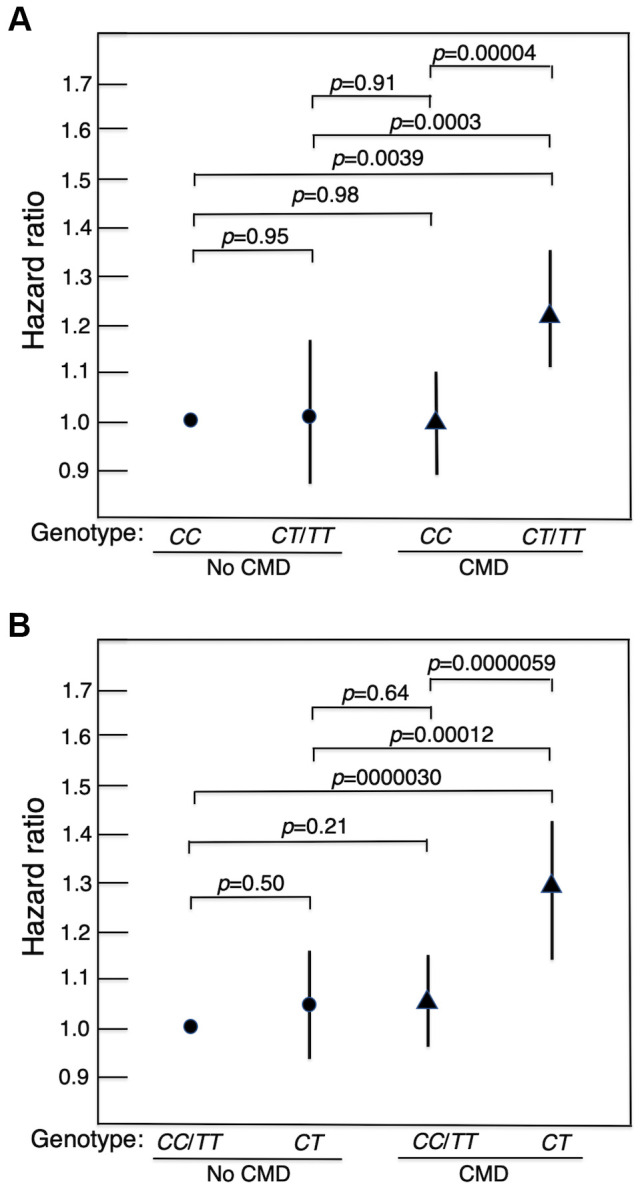
**Forest plots of mortality risk (hazard ratio and 95% CI), adjusted for age, BMI and glucose at baseline, for men with a CMD and men without a CMD according to genotype of *MAP3K5* SNP *rs2076260* in each genetic model.** (**A**) major allele homozygote (*CC*) vs. minor allele carriers (*CT*+*TT*). (**B**) heterozygote disadvantage model, *CT* vs. *CC*/*TT*. It can be seen that in men with a CMD who had the longevity-associated genotype, mortality risk was reduced to normal in that it did not differ significantly from the survival curve in men without a CMD. It should be noted that the HRs in Figure 2 differ slightly from those in Table 1. This is because the HRs in Table 1 were obtained from stratified analyses by diabetes, hypertension, CHD, and any CMD (i.e., were separately estimated by disease status). In Figure 2, we compared the HRs for the 4 groups by CMD and *MAP3K5* genotype. The HRs and *p*-values for pairwise comparisons among the 4 groups were estimated in one Cox model.

Survival curves for men without a CMD and those with a CMD showed that those who did not have a CMD lived longer (Kaplan-Meier Log-rank χ^2^ = 24.7, *p* = 1.9x10^–6^). Figure 1(A) shows survival curves for men with a CMD and men without a CMD according to whether they were major allele homozygotes (*CC*) of *rs2076260* or minor allele carriers (*CT*/*TT*). Figure 1(B) shows survival curves for the men with a CMD and for men who did not have a CMD according to whether they were heterozygous (*CT*) or homozygous (*CC*+*TT*) for each allele. These curves were determined using a Cox proportional hazard model adding an interaction term of “*MAP3K5* with CMD”. Only in men with CMD was the longevity-associated genotype associated with greater lifespan than the alternate genotype: *p* = 0.00004 for major allele homozygotes (*CC*) vs. minor allele carriers (*CC*/*TT*), and *p* = 0.000006 for heterozygotes (*CT*) vs. homozygotes (*CC*+*TT*). Overall, men without a CMD had the longest lifespans. Moreover, importantly, we noted that there was no statistical difference in lifespan for each genotype in men who did not have a CMD. Figure 2 shows forest plots of mortality risk for men with a CMD and men without a CMD for each genetic model.

**Table 1:** Hazard ratios (HR) by genotype of *MAP3K5* SNP *rs2076260* with total mortality in men with diabetes, CHD, hypertension, and any of these CMDs.

**Disease** **(n with, total)**	Cox model*	**Genetic** model**	**With a CMD**			**Without a CMD**	
			**HR (95% CI)**	***p***		**HR (95% CI)**	***p***
Diabetes	1	*CT*/*TT* vs. *CC*	1.18 (1.03–1.36)	0.016		1.11 (1.02–1.22)	0.017
(990, 2478)	2	*CT*/*TT* vs. *CC*	1.21 (1.05–1.41)	0.011		1.08 (0.98–1.19)	0.10
Hypertension	1	*CT*/*TT* vs. *CC*	1.18 (1.07–1.30)	0.0013		1.06 (0.95–1.19)	0.27
(1877, 1639)	2	*CT*/*TT* vs. *CC*	1.22 (1.09–1.36)	0.00041		1.03 (0.92–1.16)	0.60
CHD	1	*CT*/*TT* vs. *CC*	1.21 (1.02–1.42)	0.026		1.11 (1.02–1.20)	0.017
(724, 2792)	2	*CT*/*TT* vs. *CC*	1.19 (0.99–1.43)	0.059		1.11 (1.02–1.22)	0.019
Any CMD	1	*CT*/*TT* vs. *CC*	1.20 (1.09–1.31)	0.000075		0.99 (0.86–1.13)	0.83
(2461, 1055)	2	*CT*/*TT* vs. *CC*	1.23 (1.12–1.36)	0.000023		0.90 (0.78–1.05)	0.18
Diabetes	1	*CT* vs. *CC*/*TT*	1.25 (1.10–1.42)	0.00054		1.14 (1.06–1.24)	0.0010
(990, 2478)	2	*CT* vs. *CC*/*TT*	1.26 (1.10–1.44)	0.0010		1.10 (1.01–1.20)	0.029
Hypertension	1	*CT* vs. *CC*/*TT*	1.18 (1.08–1.29)	0.00043		1.14 (1.04–1.26)	0.0075
(1877, 1639)	2	*CT* vs. *CC*/*TT*	1.21 (1.09–1.34)	0.00021		1.08 (0.97–1.20)	0.18
CHD	1	*CT* vs. *CC*/*TT*	1.34 (1.15–1.55)	0.00012		1.14 (1.05–1.22)	0.0009
(724, 2792)	2	*CT* vs. *CC*/*TT*	1.33 (1.13–1.57)	0.00058		1.11 (1.02–1.21)	0.012
Any CMD	1	*CT* vs. *CC*/*TT*	1.21 (1.12–1.31)	0.0000026		1.07 (0.95–1.21)	0.25
(2461, 1055)	2	*CT* vs. *CC*/*TT*	1.22 (1.12–1.33)	0.0000081		0.98 (0.86–1.13)	0.82

*Cox models: Model 1: Age-adjusted. Model 2: Covariate-adjusted, where covariates adjusted in Cox model were: age (years), BMI (kg/m^2^), glucose (mmol/l), insulin (mIU/dL), plasma fibrinogen (mg/dl), white blood count (10^3^/μL), smoking (pack-years), alcohol intake (oz/mo), physical activity index, depression, cancer, and stroke.

**Genetic models: *Top half:* Major allele homozygote (*CC*) model. *Bottom half:* Heterozygote disadvantage model.

Age-adjusted baseline variables of men with a CMD and men without a CMD according to genotype (*CC*, *CT*, *TT*) are shown in Supplementary Table 5. None of the variables showed a difference between genotypes. The variables included body mass index (BMI), wait-to-hip ratio, fasting plasma glucose, fasting plasma insulin, plasma fibrinogen, white blood cell count, smoking, alcohol intake and physical activity index. Analyses found no evidence of population stratification or admixture in the dataset (data not shown). In men with a CMD, prevalence of diabetes, hypertension and CHD was not statistically different between each genotype.

In men with a CMD, hazard ratios (HR) for association with mortality for just diabetes, just hypertension, just CHD, and for any of these CMDs were statistically significant in two genetic models (Table 1). These were for: (1) major allele homozygotes (*CC*) vs. minor allele carriers (*CT*/*TT*), and (2) heterozygotes (*CT*) vs. homozygotes of each allele (*CC*+*TT*). No significant difference was, however, found in two other genetic models, namely for minor allele homozygotes (*TT*) vs. major allele carriers (*CC*/*CT*), and for an additive model (Supplementary Table 6). Thus, in the 1^st^ model, being homozygous for the major allele conferred strong protection against mortality in men with a CMD. In men without a CMD, however, lifespan was significantly longer irrespective of *MAP3K5* genotype. In the 2^nd^ model, heterozygotes were at a disadvantage in that men who had the *CT* genotype had a significantly higher risk of mortality than men who were homozygous for either the *C* or *T* allele.

### Functional annotations

In an attempt to determine how MAP3K5 may influence disease resistance and help to identify biological pathways we examined the following: (1) *MAP3K5* tissue expression, (2) transcription factors (TFs) that might be modified by our sentinel SNP (*rs2076260*), (3) SNPs in linkage disequilibrium (LD) that might modify transcription factor binding, (4) the expression patterns of these TFs, and (5) the location of any *cis*-regulatory elements that are physically linked with our sentinel SNP. Supplementary Figure 1 shows that *MAP3K5* is expressed in most tissues, but notably at high levels in adrenal, ovary, and pituitary.

We screened SNPs in the block of LD containing *rs2076260* (Supplementary Figure 2) for functional annotations using the HaploReg database. Seven additional SNPs were in near perfect LD with *rs2076260* and are predicted to either modify transcription factor motifs and/or are predicted to alter promoter/enhancer histone marks or DNAse I sensitivity. The latter features are associated with access to regulatory proteins. All eight SNPs were examined in more detail (Supplementary Figure 3 and Supplementary Table 7). Five of those SNPs are predicted to modify TF binding and are shown in Supplementary Table 8. Also shown is whether the minor allele is predicted to create or abolish TF binding, the biological pathways, and the tissue expression patterns. While these TFs are expressed in most tissues, they are prominent in lung and thyroid. The biological pathways include: (1) activation of immunoglobulin heavy-chain transcription, (2) repression of GATA4 and GATA6 transcription, (3) unfolded protein response, (4) activation of many muscle-specific, growth factor-induced, and stressed-induced genes, and (5) expression of metallothionein proteins in response to exposure to heavy metals.

Since the *MAP3K5* SNPs are in a non-coding region of the genome, they are presumed to affect transcription, whether directly or indirectly, as chromatin modifying units (i.e., *cis*-regulatory elements) (Supplementary Table 8). Screening of *MAP3K5* for expression quantitative loci (eQTLs) using the GTEx portal identified 1,532 entries (not shown) and four splice-site sQTLs, which are variants associated with differential splicing (not shown). Most of the eQTLs in *MAP3K5* are differentially expressed in artery (tibial), lung, breast, brain (cerebellar), esophagus (muscularis), spleen, and adipose (visceral) tissues, as defined in the GTEx database. As an aside, it should be noted that dbSNP misclassifies *rs2076260* as a mis-sense mutation (https://www.ncbi.nlm.nih.gov/projects/SNP/snp_ref.cgi?do_not_redirect&rs=rs2076260). The base change is actually in the 3^rd^ codon with no change in the encoded amino acid. We believe the confusion arose from sequencing of the opposite strand by some Human Genome Labs, where *rs2076260* is a T → C change, whereas the complement is A → G.

The longevity-associated SNP *rs2076260* and its nearest neighbor *rs6906753* in LD (D’=0.99) demonstrated significant changes in binding capacities for TFs that included homeobox D10 (HOXD10), POU class 2 homeobox 2 (POU2F2), TATA box binding protein (TBP)-associated factor, RNA polymerase II (TATA), activating transcription factor 3 (ATF3), and Hes related family BHLH transcription factor with YRPW motif 1 (HEY1) (Supplementary Figure 3). The predicted effects of putative functional variants in the SNPs on TF binding and thus tissue expression patterns are shown in Supplementary Table 9.

In order to determine whether *rs2076260* was near any additional regulatory sites and features, we examined the chromatin structures within and surrounding *MAP3K5*. Using the UCSC genome browser we identified 2 long noncoding RNAs (lncRNAs), *MAP3K5-AS1* and *LOC101928429*, located within *MAP3K5* (Supplementary Figure 4 and Supplementary Table 8)*.* From the literature we identified a site, cg21506299, that is differentially methylated in individuals with increased BMI [9]. The above entities were mapped on to the LD heatmap (Supplementary Figure 2) using the LDhap Tool (https://analysistools.cancer.gov/LDlink/?tab=ldmatrix). The locations of these features (shown in Supplementary Figure 2 and summarized in Supplementary Table 8), indicate that the adjacent longevity SNPs (asterisked in Supplementary Table 2) overlap with *MAP3K5-AS1*, that is in LD. The differentially methylated site, cg21506299, overlaps with the lncRNA *LOC101928429* near the promoter (Supplementary Figure 4).

A super-enhancer is a region of the mammalian genome comprising multiple enhancers collectively bound by an array of TFs to drive transcription of genes involved in cell identity. We identified super-enhancers overlapping the *MAP3K5* promoter, using the dbSuper database (https://asntech.org/dbsuper/index.php). These enhancers were identified in various T- and B-cell lines. They are shown in Supplementary Figure 4 and listed in Supplementary Table 10.

We mapped additional potential regulatory sites using the WashU Genome Browser. Supplementary Figure 4 shows the locations of *MAP3K5* relative to *MAP3K5-AS1*, *rs2076260,* cg21506299, *LOC101928429*, and super-enhancers*.* H3K4me3 indicates sites of tri-methylation of lysine 4 in histone H3 (green), locations of the H2 histone variant H2A.Z (blue), locations of RNA polymerase II (RNAPII) binding, and CTCF binding. H3K4me3 is associated with sites of open chromatin which are associated with activation of transcription of nearby genes [10]. H2A.Z is associated with regions of genome fluidity [10]. The *MAP3K5* promoter overlaps with H3K4me3 sites as well as H2A.Z sites and is connected, through RNAPII binding/pausing to SNP *rs2076260* as well as *MAP3K5-AS1* and *LOC101928429*. Together these features are predicted to form a *cis*-regulatory unit. This is supported by the locations of CTCF binding sites that generally form insulator domains.

## DISCUSSION

The present study has found that the longevity-associated major allele of *MAP3K5* SNP *rs2076260* is associated with protection against mortality from CMD. As a result, individuals with either one, two or all of the disorders – diabetes, hypertension or CHD – live longer if they have the protective genotype. Our study showed in fact that lifespan of men with a CMD who had the longevity-associated allele was not only longer, but did not differ significantly from lifespan of men without CMD. This indicates that possession of the *MAP3K5* longevity-associated genotype can mitigate the adverse effects on lifespan of having a CMD.

We also found an association with longevity when we tested the genetic data in a heterozygous disadvantage model. Heterozygote disadvantage is when a heterozygote has a lower overall fitness than either homozygote, and can be a potent driver of population genetic divergence [11].

Until our earlier study [7], *MAP3K5* had not previously been linked directly to human aging and longevity. Most studies have, however, involved European populations [10]. According to dbSNP, the frequency of the *C* allele of *rs2076260* in the 1000 Genomes East Asian super-population is 0.55, whereas it is only 0.17 in the European super-population (https://www.ncbi.nlm.nih.gov/snp/?term=rs2076260), thus diminishing the chance of detection of an association with longevity in genetic association studies of Europeans. Various genetic factors have been identified that are associated with aging-related risk factors and diseases [12]. In Japanese people, healthy traditional dietary, lifestyle and cultural factors, by reducing risk of CMDs, are thought to contribute to their longer average lifespans [13]. In extreme old age genetic factors become more important than environmental factors in lifespan determination [10]. Our study used a homogeneous population of American men of Japanese ancestry in which there was no evidence of population stratification or admixture [14]. Thus, various favorable factors appear to have contributed to our previous positive genetic finding of an association of SNPs in *MAP3K5* with longevity [7].

MAP3K5 is involved in stress response, inflammation, and apoptosis [1]. Disruption of *Map3k5* in mice attenuates left ventricular remodelling [15]. Prolonged activation of p38 or JNK by MAP3K5 results in long-term cellular damage. MAP3K5 inhibition may serve as a therapeutic target, as summarized in studies that have tested the effects of MAP3K5/ASK1 inhibition in cell and animal disease models, as well as in human clinical trials for a variety of diseases [16].

*MAP3K5* is expressed in a large number of tissues, most notably adipose tissue, artery, Epstein barr virus-transformed lymphocytes, ovary, and pituitary (as documented in the GTEx database: https://gtexportal.org/home/gene/MAP3K5). Moreover, MAP3K5 has several isoforms that are differentially expressed in various tissues (see GTEx database). It is likely that the longevity-associated *MAP3K5* genotype is in LD with functional allele(s) of regulatory variant(s) of *MAP3K5* that have a stronger effect on gene expression than the alternative genotypes, so boosting the levels of the encoded MAP3K5 protein and leading to higher beneficial biological effects, such as apoptosis. Two longevity-associated SNPs, *rs2076260* and its near neighbor *rs6904753*, in LD with each other, are predicted to influence binding capacities for six transcription factors. The common allele of *rs2076260* is predicted to create/enhance binding of factors HOXD10, POU2F2, and TATA, thus increasing *MAP3K5* expression, but reduce binding of DMRT1, while the common allele of *rs6904753* is predicted to abolish/reduce binding of factors ATF3 and HEY1, which are generally repressive, and thus increase *MAP3K5* expression. The additional SNPs in LD may also influence TF binding. Since these TFs are differentially expressed, their specific effect would depend on the tissue.

Data on the tissue expression distributions for the lncRNAs were not available. The lncRNA, *MAP3K5-AS1*, located within *MAP3K5* close to *rs2076260*, may influence *MAP3K5* expression, either negatively by recruiting RNAPII on the negative strand or positively by recruiting other transcription enhancers and/or splicing factors on the positive strand. Since *MAP3K5-AS1* is transcribed in the opposite direction as *MAP3K5*, it operates in effect as an anti-sense RNA, interfering with *MAP3K5* transcription, as above. The possibility of a role for *MAP3K5-AS1* in the genotypic effect we have observed is therefore of interest. The cg21506299 site differentially methylated in subjects with increased BMI [9], and located in the *MAP3K5* promoter, may be of interest in mediating the contrasting genotypic effect found in our men who had a CMD. This is because high BMI is associated with diabetes, hypertension and CHD. Another lncRNA, *LOC101928429*, at this site may influence transcription as well. Notable too was that the promoter and both lncRNAs overlap with peaks of H3K4me3 tri-methylation and of peaks of the histone variant H2A.Z, which is associated with regions of genome fluidity in cells [10].

In conclusion, in two closely related genetic models, we show that longevity-associated genotypes of *MAP3K5* are associated with virtually complete mitigation of the lifespan shortening effect of having a CMD. Since lifespan was the same irrespective of genotype in men without a CMD, the overall association of genetic variation in *MAP3K5* with longevity is contributed entirely by allele-dependent amelioration of the increased mortality risk conferred by CMD. This finding adds to our recent findings of a similar explanation for the longevity association conferred by particular SNPs in *FOXO3* [17] and *PIK3R5* [18]. The effect does not, however, explain the longevity association we have found for most other genes in our cohort of elderly men [7, 19] (unpublished).

## MATERIALS AND METHODS

### Study participants

See Supplementary Methods and Supplementary Table 1.

### Genotyping

Genotyping methods were as described previously [7] (Supplementary Methods).

### Variant search

Variants surrounding *rs2076260* were screened on the RegulomeDB site, which includes known and predicted regulatory elements in the intergenic regions, as well as regions of DNAase hypersensitivity, binding sites for transcription factors, and promoter regions. Sources of these data included public datasets from GEO, the ENCODE project, and published literature [20]. Chromosome 6 locations used the GRCh37.p13 genome build (http://www.gencodegenes.org/releases/19.html).

We also screened the variants using HaploReg, which is a tool for exploring annotations of the noncoding genome at variants on haplotype blocks, such as candidate regulatory SNPs at disease-associated loci [21]. Using LD information from the 1000 Genomes Project, linked SNPs can be visualized along with chromatin state and protein binding annotation from the Roadmap Epigenomics and ENCODE projects, the effect of SNPs on regulatory motifs, and the effect of SNPs on expression from QTL studies. We searched HaploReg version 4.1 for the region between SNPs in LD, *rs9494547* and *rs9494552*, Nov 5, 2015 build, hg38.

### Statistical analyses

General linear models were used to compare age-adjusted indirect measurements between groups, and logistic models were used to compare the age-adjusted direct measurements. Cox proportional models were used to assess the association of *MAP3K5* for various genetic models on mortality stratified by disease status, such as by diabetes, by hypertension, by CHD, and by any of CHD, diabetes, or hypertension. The Cox proportional hazard assumption was tested for each Cox model. The effect of interaction of disease with *MAP3K5* genotype on mortality was tested in the Cox model. All statistical analyses were performed using the Statistical Analysis System version 9.4 [22]. Figures were generated using STATA 12 Graphics [23].

## Supplementary Material

Supplementary Materials and Methods

Supplementary Figures

Supplementary Tables
