# Supplementary material for: Lifespan extension conferred by mitogen-activated protein kinase kinase kinase 5 (MAP3K5) longevity-associated gene variation is confined to at-risk men with a cardiometabolic disease
Source: Aging (Albany NY). 2021 Mar 19;13(6):7953–74. doi: 10.18632/aging.202844 (PMC8034933; doi:10.18632/aging.202844)
Supplement: Supplementary Figures [file aging-13-202844-s002.pdf]

SUPPLEMENTARY FIGURES

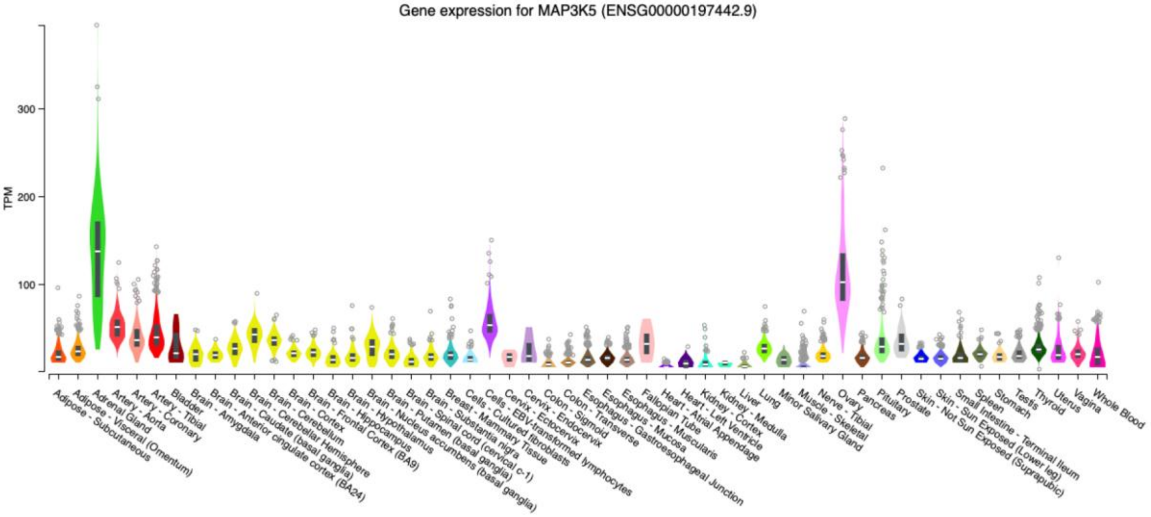

Supplementary Figure 1. Tissue expression pattern of *MAP3K5*. This gene is expressed at high levels in most tissues, most notably in adrenal, artery, ovary, and pituitary. A value of 50 TPM (transcripts per million) is arbitrarily chosen as cut-off for significance. TPM is a measurement of the proportion of transcripts in the pool of RNA, as measured by RNA-seq.

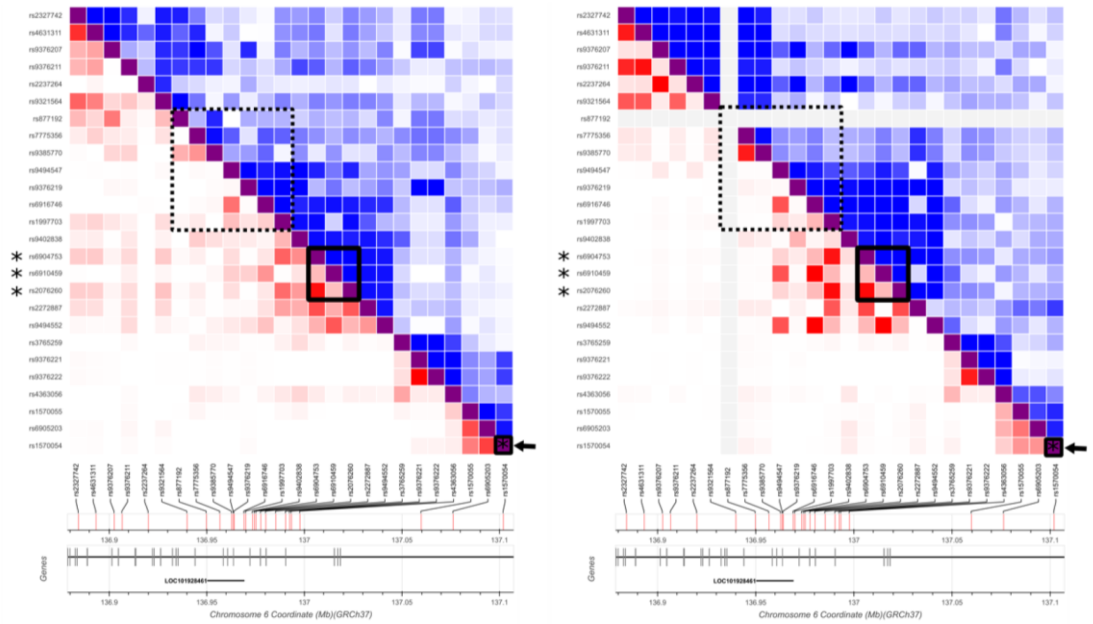

Supplementary Figure 2. Heatmap of the locations of the longevity-associated SNPs in our original study [11]. Also shown is the location of the differentially methylated site reported by Horsburgh et al. [12] (asterisk), and the location of two long non-coding RNAs (lncRNA), *MAP3K5-AS1* (previously *LOC101928461*) within *MAP3K5* (dotted box), and *LOC101928429* (small solid box near promoter and asterisk). Both lncRNAs are transcribed in the direction opposite (left to right) to that of *MAP3K5* and are referred to as anti-sense RNAs. They may act to interfere with transcription. *MAP3K5* is transcribed from right to left (3' to 5'). *MAP3K5-AS1* overlaps with the block of LD. *MAP3K5-AS1* is in LD with the longevity-associated SNPs we identified previously in *MAP3K5*. Little is, however, known about the lncRNA; the precise location is close to but not exactly the same as the differentially spliced isoform of *MAP3K5*. The LD map for the Japanese population (JPT) is shown in on the left, while that for the Caucasian population (CEU) is shown on the right.

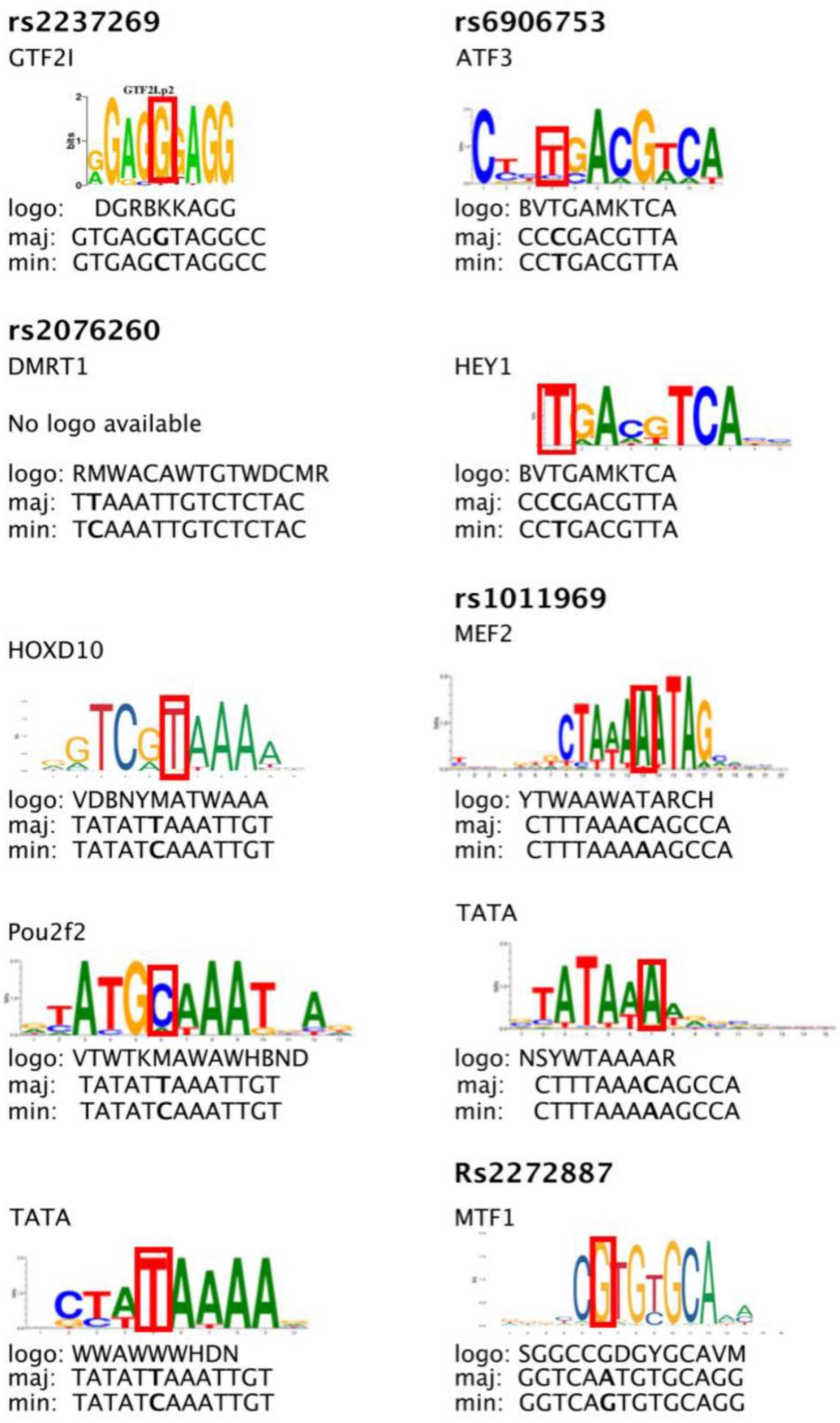

Supplementary Figure 3. Transcription factor binding sites modified by SNP *rs2076260*. *rs2076260* and its immediate neighboring SNP, *rs6904753*, are in perfect LD. SNP *rs2076260* is predicted to modify the binding of three transcription factors, HOXD10, Pou2f2, and TATA. The major allele is predicted to create/increase binding of HOXD10, abolish/reduce binding of Pou2f2, and create/increase binding of TATA. For *rs6906753* the major allele would reduce/abolish binding of ATF3 and HEY1 transcription factors. Red rectangles denote the variant SNP nucleotide in the transcription factor canonical sequence. (Abbreviations: maj, major allele; min, minor allele; logo is the canonical recognition site for each. Nucleotide ambiguity codes are: B, not A; V, not T; M, C or A; K, T or G; W, A or T).

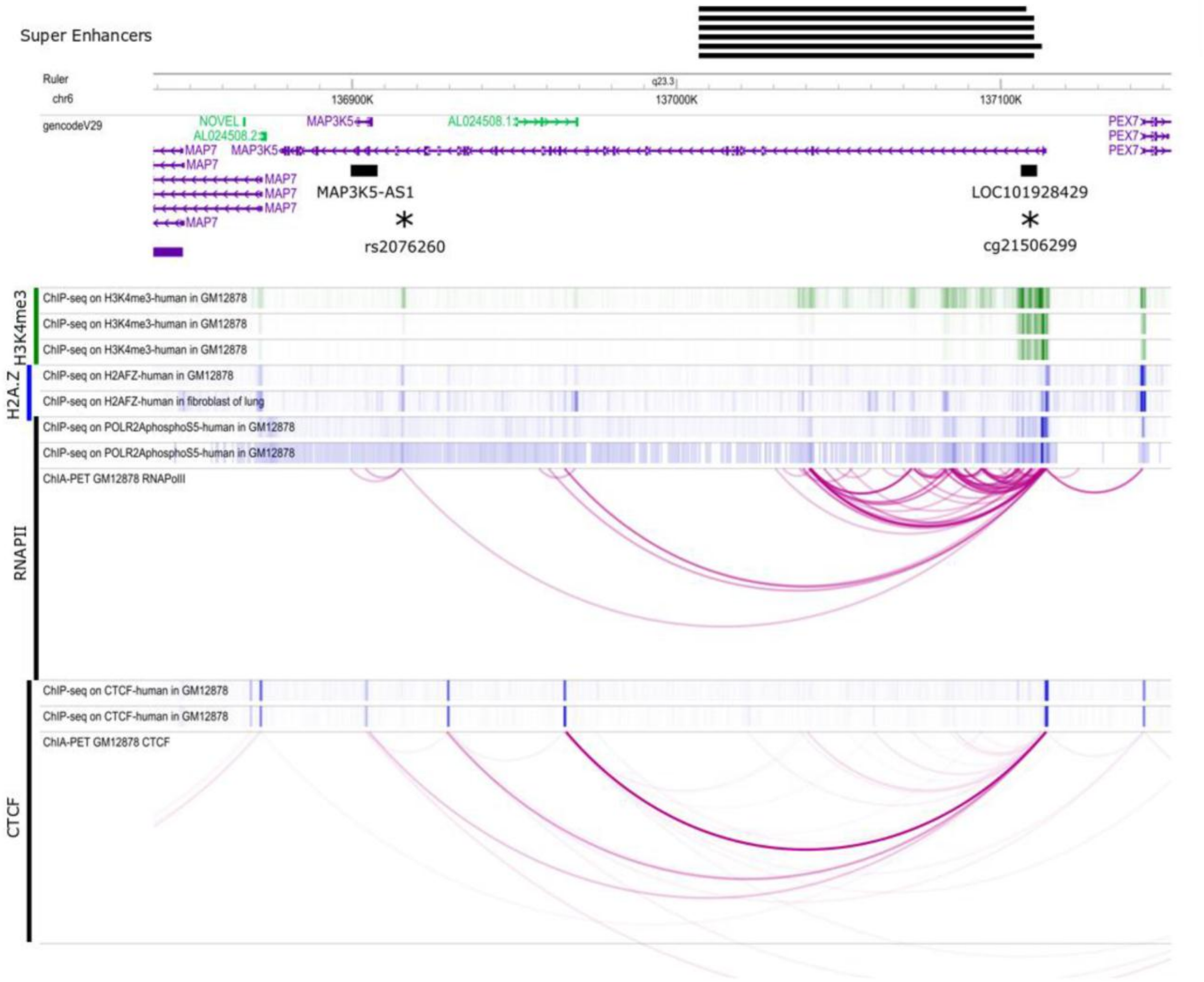

Supplementary Figure 4. Possible functional relationships between *rs2076260* and regulatory features in *MAP3K5*. We postulate that *rs2076260* interacts with other *cis*-regulatory elements. Shown by asterisks are the locations of the most significant SNP (*rs2076260*) in *MAP3K5* relative to two long non-coding RNAs, *MAP3K5-AS1* and *LOC101928429*, and the differentially methylated site associated with BMI, cg21506299. The anti-sense lncRNAs as well as SNP *rs2076260* and the promoter proper overlap with peaks of H3K4me4 (green) (see review by Morris et al. [13]], which is commonly associated with sites of activation of transcription of nearby genes, the histone variant H2A.Z (blue), which is associated with regions of genome fluidity (see review Morris et al. [13]). GM12878 (lymphocyte) experiments were chosen for reference. These locations are connected via RNA polymerase II (RNAPolII) and CTCF chromatin looping, indicating that they are likely co-regulated during transcription. *MAP3K5* is transcribed from right to left (purple herring bone). GM12878 (lymphocyte) experiments were chosen for reference. The locations of super-enhancers are shown by solid bars at top.
