# Supplementary material for: Lifespan extension conferred by mitogen-activated protein kinase kinase kinase 5 (MAP3K5) longevity-associated gene variation is confined to at-risk men with a cardiometabolic disease
Source: Aging (Albany NY). 2021 Mar 19;13(6):7953–74. doi: 10.18632/aging.202844 (PMC8034933; doi:10.18632/aging.202844)
Supplement: Supplementary Tables [file aging-13-202844-s003.pdf]

SUPPLEMENTARY TABLES

Supplementary Table 1. Characteristics of cases (n=440) and controls (n=374), age-adjusted (except age and year of birth) used in our prior case-control studies.

Late life examination characteristics	Controls	Cases	*p*
Age at examination, y	74.7 ± 2.1	81.5 ± 4.7	2.1E–107*
Birth year	1916.8 ± 2.0	1909.9 ± 4.7	1.9E–111*
Anthropometric and physiological			
Height, cm	161.2 ± 5.6	161.3 ± 5.5	0.96
Weight, kg	59.6 ± 9.6	61.6 ± 8.0	0.020
Waist to hip ratio	0.95 ± 0.06	0.94 ± 0.05	0.015
BMI, kg/m^2	23.0 ± 3.1	23.6 ± 2.9	0.040
Triceps skinfold thickness, mm	10.5 ± 4.1	10.2 ± 4.1	0.40
Subscapular skinfold thickness, mm	15.5 ± 6.7	16.5 ± 5. 5	0.09
Best forced expiratory volume in 1 s, L	1.9 ± 0.49	2.1 ± 0.41	1.5E–06*
Grip strength, kg	26.9 ± 7.1	31.7 ± 5.2	1.2E–15*
Blood pressure, systolic, mmHg	151.0 ± 26.3	150.4 ± 22.4	0.079
Blood pressure, diastolic, mmHg	79.0 ± 12.7	81.6 ± 11.6	0.022
Ankle-brachial index	1.0 ± 0.20	1.1 ± 0.14	2.1E–14*
Cognitive (CASI) score	75.6 ± 19.8	87.1 ± 10.6	2.2E–5*
Hematological and biochemical			
Total cholesterol, mg/dL	183 ± 34.2	195 ± 32.6	0.00034*
HDL cholesterol, mg/dL	51.3 ± 14.4	51.3± 13.1	0.97
Triglycerides, mg/dL	146.5 ± 119.3	152.3 ± 78.7	0.54
Fasting plasma glucose, mg/dL	118.5 ± 36.8	108.6 ± 21.5	0.00054*
Fasting plasma insulin, mIU/dL	26.2 ± 84.3	13.1 ± 9.5	0.018
Plasma fibrinogen, mg/dL	331 ± 75.8	291 ± 52.7	1.5E–10*
White blood cell count, 10^3/µL	6.98 ± 2.09	5.87 ± 1.50	1.3E–10*
Health habits			
Current smoker, %	13.6	4.0	0.00035*
Past smoker, %	60.0	48.3	0.016
Smoking, pack-years	37.2 ± 35.8	17.4 ± 30.5	2.9E–9*
Alcohol consumption, ounces/month	29.4 ± 53.6	14.4 ± 29.2	0.00033*
Physical activity index, metabolic work/day	29.9 ± 4.6	31.7 ± 4.4	3.5E–5*
Difficulty in walking 0.8 km, %	39.8	6.6	1.0E–17*
On diabetes medication, %	18.1	6.9	0.00026*
Diseases			
Hypertension, %	74.1	78.8	0.24
Coronary heart disease, %	26.7	14.2	0.00085
Stroke history, %	9.3	1.2	7.6E–5*
Cancer, %	23.8	10.0	8.3E–5*
Diabetes, %	35.0	21.7	0.00011*
Emphysema, %	4.8	1.4	0.038
Bypass history, %	11.9	3.5	0.05
Angioplasty, %	4.1	1.4	0.37
Surgery on arteries of the neck, %	1.4	0.69	0.41
Aorta surgery, %	8.4	0.92	8.7E–7*
Ankle-brachial index < 0.9. %	24.8	3.7	6.9E–11*
Sociodemographic			
Education, years	10.2 ± 2.8	10.5 ± 3.5	0.30
Married, %	75.5	86.9	0.002

The data shown are age-adjusted. *Indicates that difference remained significant after the *p* value shown was adjusted by the Bonferroni method for the 41 parameters tested.

Supplementary Table 2. The SNPs genotyped in *MAP3K5* and the minor allele frequency of each in control American men of Japanese ancestry in the Kuakini Honolulu Heart Program, and of Japanese subjects in the dbSNP database.

SNP	KHHP	dSNP
rs1570055	0.479	0.488
rs1570054	0.445	0.465
rs12529435	0.181	0.133
rs2327742	0.546	0.488
rs9376222	0.103	0.111
rs7775356	0.297	0.278
rs6905203	0.433	0.442
rs877192	0.256	0.193
rs6910459	0.238	0.221
rs1997703	0.418	0.407
*rs2076260**	0.455	0.458
rs12164028	0.345	0.343
rs17723504	0.696	0.200
rs9376207	0.233	0.144
*rs6904753**	0.467	0.477
rs9385770	0.479	0.447
rs17796708	0.252	0.244
rs4631311	0.383	0.378
rs6916746	0.118	0.110
rs2272887	0.483	0.477
rs9494547	0.171	0.169
rs13203080	0.241	0.221
rs2237264	0.172	0.148
rs9376211	0.173	0.278
rs13195420	0.189	0.140
rs3765259	0.441	0.401
rs9402838	0.270	0.364
rs9376219	0.183	0.134
rs9494552	0.284	0.273
rs9321564	0.480	0.424
rs4363056	0.409	0.471
rs17723638	0.189	0.156
rs9376221	0.103	0.099

*Denotes those SNPs in a cluster associated with longevity using a dominant model. A cluster was defined as SNPs in close physical proximity and/or belonging to the same block of linkage disequilibrium (LD).

Supplementary Table 3. The two *MAP3K5* SNPs in the case-control study that were associated with longevity after Bonferroni correction and results for the four genetic models used.

SNP	Estimate	S.E.	OR	*p*	Model*	C_H	*p*	R_H	*p*
rs2076260	–0.079	0.098	0.92	0.42	Additive	–	–	–	–
rs2076260	–0.637	0.151	0.53	<.0001	Het vs other	1.95	0.0001	1.81	0.0018
rs2076260	0.4527	0.159	1.57	0.0043	MM vs other	–	–	–	–
rs2076260	0.2987	0.174	1.35	0.086	RR vs other	–	–	–	–
rs6904753	–0.066	0.098	0.936	0.50	Additive	–	–	–	–
rs6904753	–0.417	0.141	0.659	0.0032	Het vs other	1.57	0.0066	1.45	0.040
rs6904753	0.336	0.157	1.40	0.032	MM vs other	–	–	–	–
rs6904753	0.202	0.170	1.22	0.23	RR vs other	–	–	–	–

OR was estimated using a logistic model with long-lived case as the outcome. If OR was significantly > 1, then the genotype comparison indicated a pro-longevity effect.
*Model:
Additive model: gene effect is coded as common homozygote (*CC*) = 0, heterozygote (*CT*) =1, minor allele homozygote (*TT*) = 2 in a linear logistic model.
Het = heterozygote (*CT*).
MM = common homozygote (*CC*), and RR = minor allele homozygote (*TT*).
C_H: OR of common homozygote (*CC*) vs. heterozygote (*CT*) with long-lived case.
R_H: OR of minor allele homozygote (*TT*) vs. heterozygote (*CT*) with long-lived case.
Other: The other genotype(s).
S.E. = standard error of the mean.

Supplementary Table 4. Characteristics of all subjects at baseline (1991–1993) by *MAP3K5 rs2076260* genotype.

Characteristics	*CC*	*CT*	*TT*	*p*
**n**	1000	1733	783	
Age at examination, yr	77.8 ± 4.6	77.6 ± 4.6	77.8 ± 4.7	0.48
Birth year	13.6 ± 4.6	13.8 ± 4.6	13.7 ± 4.7	0.48
**Anthropometric and physiological**				
Height, cm	161.9 ± 5.7	161.7 ± 5.5	161.6 ± 5.9	0.66
Weight, kg	61.7 ± 9.4	61.2 ± 8.9	61.4 ± 9	0.35
Waist to hip ratio	0.9 ± 0.1	0.9 ± 0.1	0.9 ± 0.1	0.98
BMI, kg/m^2	23.6 ± 3.2	23.4 ± 3	23.5 ± 3.1	0.31
Triceps skinfold thickness, mm	10.1 ± 4	10.1 ± 3.9	10.1 ± 4	0.90
Subscapular skinfold thickness, mm	16.3 ± 6.1	16 ± 5.9	16.4 ± 6.5	0.36
Best forced expiratory volume	2.1 ± 0.5	2.1 ± 0.4	2.1 ± 0.5	0.58
Grip strength, kg	30.4 ± 5.8	30.1 ± 6.2	30.2 ± 6.2	0.55
Blood pressure, systolic, mmHg	148.9 ± 23.9	149.5 ± 23.5	149.5 ± 22.8	0.83
Blood pressure, diastolic, mmHg	79.9 ± 11.3	79.8 ± 11.6	80.3 ± 10.7	0.60
Cognitive (CASI) score	83.3 ± 13.9	82.2 ± 15.3	82.9 ± 13.4	0.14
**Hematological and biochemical**				
Total cholesterol, mg/dL	191.1 ± 32.2	188.9 ± 32.8	190.3 ± 33.3	0.24
HDL cholesterol, mg/dL	50.7 ± 13.6	51.1 ± 13.1	51 ± 13.5	0.76
Triglycerides, mg/dL	151.2 ± 97.7	147.7 ± 91.5	149.3 ± 95.2	0.63
Fasting plasma glucose, mg/dL	113.9 ± 29.7	113.2 ± 30.4	111.5 ± 27	0.24
Fasting plasma insulin, mIU/dL	15.9 ± 14.7	15.4 ± 13.1	15.3 ± 11.7	0.56
Plasma fibrinogen, mg/dL	305.8 ± 65.8	306.9 ± 63.3	308.4 ± 63.3	0.71
White blood cell count, 10^3/µL	6.3 ± 2.5	6.3 ± 1.7	6.3 ± 2.4	0.96
**Health habits**				
Current smoker, %	6.5	7	7.3	0.80
Past smoker, %	55.1	56.3	53	0.33
Smoking, pack-years	26.1 ± 34.4	26.9 ± 34.2	23.6 ± 33	0.10
Alcohol consumption, ounces/month	18.7 ± 39.1	18.8 ± 39.9	18.9 ± 43	1.00
Physical activity index, metabolic work/day	31 ± 4.7	30.8 ± 4.5	30.9 ± 4.7	0.55
Difficulty in walking 0.8 km, %	19.4	18.1	18.3	0.65
On diabetes medication, %	11.4	11.6	10.1	0.51
**Diseases**				
Hypertension (160/95), %	54.2	53.2	52.9	0.83
Coronary heart disease, %	20	20.6	21.3	0.79
Stroke history, %	3.6	4.9	3.9	0.25
Cancer, %	11.7	13.7	14.9	0.13
Diabetes, %	28.9	28.3	28.6	0.94
Emphysema, %	2.7	3.2	2.2	0.40
Bypass history, %	7.6	7.7	5.8	0.19
Angioplasty, %	6	7.3	6.6	0.40
Surgery on arteries of the neck, %	1.3	1.1	0.9	0.74
Aorta surgery, %	3	3.7	2.8	0.40
Ankle-brachial index < 0.9, %	12.3	12.6	12.5	0.96
**Sociodemographic**				
Education, years	10.6 ± 3.1	10.5 ± 3.1	10.6 ± 3.2	0.53
Married, %	85.5	82.9	80.7	0.029

Supplementary Table 5. Age-adjusted baseline variables by cardiometabolic disease (CMD)* status and *MAP3K5 rs2076260* genotype.

Variable	With CMD*				Without CMD			
	CC	*CT*	*TT*	*p***	*CC*	*CT*	*TT*	*p*
**n**	701	1212	548		299	521	235	–
Age (years)	77.9 ± 4.6	77.6 ± 4.5	77.9 ± 4.6	0.35	77.7 ± 4.5	77.6 ± 4.7	77.5 ± 4.7	0.82
BMI (kg/m²)	23.8 ± 3.2	23.6 ± 3	23.7 ± 3.1	0.61	23 ± 3	22.7 ± 3	23.1 ± 3.1	0.20
Waist to hip ratio	0.95 ± 0.06	0.95 ± 0.06	0.95 ± 0.06	0.60	0.94 ± 0.06	0.93 ± 0.06	0.94 ± 0.05	0.34
Fasting plasma glucose (mg/dL)	119.2 ± 33.6	118.3 ± 34.7	116 ± 30.8	0.20	101.3 ± 8.4	101.2 ± 8.7	101.5 ± 8.5	0.92
Fasting plasma insulin (mIU/dL)	20.1 ± 49.6	17.9 ± 24.3	17.7 ± 29.1	0.33	12.1 ± 6.8	12 ± 7	12.5 ± 6.8	0.62
Plasma fibrinogen (mg/dL)	309.2 ± 65.7	309.3 ± 63.9	314.1 ± 66.5	0.32	297.8 ± 65.4	301.5 ± 61.5	295.1 ± 53	0.38
White blood cell count (10^3/μL)	6.4 ± 2.8	6.4 ± 1.7	6.4 ± 2.6	0.96	5.9 ± 1.7	6 ± 1.7	5.9 ± 1.6	0.81
Smoking (pack-years)	27.5 ± 35.6	27.3 ± 34.2	24.6 ± 34.3	0.28	22.8 ± 31.1	26.0 ± 34.1	21.5 ± 29.8	0.18
Alcohol intake (oz/mo)	19.2 ± 39.8	20.8 ± 44.9	18.9 ± 43.6	0.64	17.7 ± 37.4	14.3 ± 24.3	18.8 ± 41.5	0.18
Physical activity index	30.9 ± 4.7	30.7 ± 4.5	30.8 ± 4.7	0.60	31.1 ± 4.5	31.0 ± 4.4	31.1 ± 4.6	0.91
Depression (%)	9.1	10	12.4	0.18	11.8	10.5	10.1	0.82
Stroke (%)	4.5	5.6	4.7	0.51	1.7	3.1	2.2	0.39
Cancer (%)	12.8	12.8	15.3	0.31	9.2	15.9	14	0.026
Diabetes (%)	41.2	40.4	41.0	0.94	–	–	–	–
Hypertension (%)	77.3	76	75.5	0.74	–	–	–	–
CHD (%)	99.9	99.9	100	0.120	–	–	–	–

Values shown are mean ± SD for indirect measures and proportion (%) for direct measurements.
*CMD: diabetes or hypertension or CHD.
***p* from Fishers F-test.

Supplementary Table 6. Hazard ratios (HR) by genotype of *MAP3K5* SNP *rs2076260* with total mortality in men with diabetes, CHD, hypertension, and any of these CMDs in two other genetic models besides the two models shown in Table 1 of the main article.

Disorder (n with, total)	Cox model*	Genetic model**	With a CMD		Without a CMD	
			HR (95% CI)	*p*	HR (95% CI)	*p*
Diabetes	1	*CC*/*CT* vs. *TT*	1.11 (0.96-1.29)	0.17	1.06 (0.97-1.17)	0.2091
(990, 2478)	2	*CC*/*CT* vs. *TT*	1.08 (0.92-1.27)	0.34	1.05 (0.94-1.16)	0.4160
Hypertension	1	*CC*/*CT* vs. *TT*	1.03 (0.93-1.15)	0.56	1.12 (1.00-1.26)	0.0515
(1877, 1639)	2	*CC*/*CT* vs. *TT*	1.02 (0.90-1.15)	0.79	1.08 (0.95-1.23)	0.2517
CHD	1	*CC*/*CT* vs. *TT*	1.19 (1.00-1.42)	0.050	1.06 (0.97-1.16)	0.1866
(724, 2792)	2	*CC*/*CT* vs. *TT*	1.19 (0.98-1.45)	0.071	1.02 (0.92-1.13)	0.7077
Any CMD	1	*CC*/*CT* vs. *TT*	1.06 (0.96-1.16)	0.25	1.13 (0.97-1.31)	0.11
(2461, 1055)	2	*CC*/*CT* vs. *TT*	1.03 (0.92-1.14)	0.63	1.10 (0.93-1.30)	0.26
Diabetes	1	Additive	1.03 (0.95-1.12)	0.48	1.02 (0.97-1.08)	0.43
(990, 2478)	2	Additive	1.04 (0.95-1.14)	0.35	1.02 (0.96-1.08)	0.53
Hypertension	1	Additive	1.06 (0.99-1.12)	0.087	0.98 (0.92-1.05)	0.65
(1877, 1639)	2	Additive	1.08 (1.00-1.15)	0.036	0.98 (0.91-1.06)	0.61
CHD	1	Additive	1.01 (0.92-1.11)	0.82	1.02 (0.97-1.07)	0.45
(724, 2792)	2	Additive	1.00 (0.90-1.11)	0.98	1.04 (0.98-1.10)	0.20
Any CMD	1	Additive	1.05 (1.00-1.11)	0.065	0.95 (0.88-1.04)	0.28
(2461, 1055)	2	Additive	1.08 (1.01-1.14)	0.015	0.93 (0.84-1.02)	0.14

*Cox models: Model 1: Age-adjusted. Model 2: Covariate-adjusted, where covariates adjusted in Cox model were: age, BMI, glucose, smoking (pack-year), alcohol intake (oz/mo), physical activity index, depression, cancer, and stroke.
**Genetic models: *Top half:* Minor allele homozygote model. *Bottom half:* Additive model, in which the genetic variables were coded as 0 for genotype *CC*, 1 for *CT* and 2 for *TT*, so that HR represents the additive effect of genotype on mortality.

Supplementary Table 7. Variants in near perfect linkage disequilibrium (LD) with *rs2076260*, and additional information for each.

chr	pos (hg19)	LD (r^2)	LD (D')	Variant	Ref/ Alt	ASN freq	EUR freq	Promoter histone marks	Enhancer histone marks	DNAse	Motifs changed
6	136966619	0.81	0.98	*rs2237269*	G/C	0.37	0.16			HRT, BLD	4 altered motifs
6	136966741	0.93	0.98	*rs2237270*	T/C	0.37	0.17			–	
6	136968541	0.94	0.99	*rs2237271*	A/C	0.45	0.17	6 tissues	17 tissues	20 tissues	
6	136969853	0.97	1.00	*rs1997703*	C/T	0.45	0.18		7 tissues	–	
6	136974283	0.97	0.99	*rs6904753*	C/T	0.55	0.18		ADRL	–	4 altered motifs
6	136977568	1.00	1.00	*rs2076260*	T/C	0.56	0.18		6 tissues	MUS, BRN	6 altered motifs
6	136979240	0.84	1.00	*rs1011969*	C/A	0.55	0.16			GI	Mef2, TATA
6	136980134	0.83	0.99	*rs2272887*	A/G	0.48	0.16		ESDR, BRN, MUS	5 tissues	Mtf1

All positions are based on genome release GRCh37 released Feb 2009 (UCSC equivalent = hg19).
Abbreviations: ADRL, adrenal; ESDR, embryonic stem cell derived; BLD, blood; BRN, brain; GI, gastrointestinal; HRT, heart; MUS, muscle.

Supplementary Table 8. Summary of nucleotide sequences and putative functional features for *MAP3K5.*

Feature	Location (hg19)	Definition
MAP3K5	136,977,568–137,105,225	Gene (transcribed right to left, i.e., 3'–5')
MAP3K5-AS1	136,950,252–136,969,336	LncRNA (transcribed left to right, 5'–3')
rs2076260	136,977,568	Longevity-associated SNP, this study
rs6904753	136,974,283	Neighboring functional SNP
LOC101928429	137,105,185-137,107,192	LncRNA (transcribed left to right, 5'–3')
cg21506299	137,105,225	Site differentially methylated with BMI

*hg19, the genome build describing where features are located. The actual sequences from NCBI/UCSC/Ensembl are identical to these, but their annotations will be different and updated at different frequencies.

Supplementary Table 9. Putative functional variants and their effects on transcription of the common allele.

SNP	Result (minor allele)	TF	Biological pathways	Tissue expression*
rs2237269	abolish	*GTF2I*	Activation of immunoglobulin heavy-chain transcription upon B-lymphocyte activation	Most tissues
rs6904753	create	*HEY1*	Repressive, induced by the Notch and c-Jun, repressive for GATA4 and GATA6	Lung
	create	*ATF3*	Unfolded protein response, stress response activating or repressive	Lung, thyroid
rs2076260	create	*DMRT1/7*	Sex-determining pathway	< 100 FPKM
	abolish	*HOXD10*	Developmental regulatory system for anterior-posterior axis.	< 100 FPKM
	abolish	*POU2F2*	Immunoglobulin gene promoter,	< 100 FPKM
	abolish	*TATA*	RNA polymerase II initiation	< 100 FPKM
rs1011969	create	*MEF2*	Activates many muscle-specific, growth factor-induced, and stress-induced genes	Most tissues
	create	*TATA*	Essential for progression of the G1 phase of the cell cycle	Most tissues
rs2272887	create	*MTF1*	Induces expression of metallothioneins in response to exposure to heavy metals	Most tissues

Data from HaploReg, v4.1. Biological pathway prediction is from GeneCards. Tissue expression patterns are from GTEx.
*< 100 FPKM, fragments per kilobase of transcript per million mapped reads (indicates low level of expression).

Supplementary Table 10. Super-enhancers overlapping *MAP3K5*, and their chromosomal location, size and tissue.

ID	Chrom	Start	End	Size	Associated gene	Method	Rank	Cell/tissue
SE_32506	chr6	137070606	137115431	44825	*MAP3K5*	H3K27ac	52	GM12878
SE_59682	chr6	137063070	137114568	51498	*MAP3K5*	H3K27ac	79	Ly4
SE_09572	chr6	137071400	137114901	43501	*MAP3K5*	H3K27ac	436	CD14
SE_11351	chr6	137070304	137115433	45129	*MAP3K5*	H3K27ac	508	CD20
SE_10763	chr6	137071728	137113378	41650	*MAP3K5*	H3K27ac	608	CD19 Primary
SE_19826	chr6	137071508	137115095	43587	*MAP3K5*	H3K27ac	728	CD4p CD25- Il17p PMAstim Th17

Data are from dbSUPER, (https://asntech.org/dbsuper/index.php) positions are according to GRCh37/hg19.
ID refers to dbSUPER identification number. For descriptions of methods and rank see Hnisz et al. [14].
